# Cellular differentiation determines the expression of the hypoxia-inducible protein NDRG1 in pancreatic cancer

**DOI:** 10.1038/sj.bjc.6603256

**Published:** 2006-07-11

**Authors:** E Angst, S Sibold, C Tiffon, R Weimann, B Gloor, D Candinas, D Stroka

**Affiliations:** 1 Department of Clinical Research, Visceral and Transplant Surgery, University Hospital Bern, Murtenstrasse 35, Bern 3010, Switzerland; 2 Institute of Pathology, University of Bern, Bern 3010, Switzerland

**Keywords:** pancreatic cancer, NDRG1, cellular differentiation, hypoxia

## Abstract

N-myc downstream-regulated gene-1 (NDRG1) is a recently described hypoxia-inducible protein that is upregulated in various human cancers. Pancreatic ductal adenocarcinoma, called pancreatic cancer, is a highly aggressive cancer that is characterised by its avascular structure, which results in a severe hypoxic environment. In this study, we investigated whether NDRG1 is upregulated in these tumours, thus providing a novel marker for malignant cells in the pancreas. By immunohistochemistry, we observed that NDRG1 was highly expressed in well-differentiated cells of pancreatic cancer, whereas the poorly differentiated tumour cells were negative. In addition, hyperplastic islets and ducts of nonquiescent pancreatic tissue were positive. To further explore its selective expression in tumours, two well-established pancreatic cancer cell lines of unequal differentiation status were exposed to 2% oxygen. NDRG1 mRNA and protein were upregulated by hypoxia in the moderately differentiated Capan-1 cells; however, its levels remained unchanged in the poorly differentiated Panc-1 cell line. Taken together, our data suggest that NDRG1 will not serve as a reliable marker of tumour cells in the pancreas, but may serve as a marker of differentiation. Furthermore, we present the novel finding that cellular differentiation may be an important factor that determines the hypoxia-induced regulation of NDRG1.

Pancreatic ductal adenocarcinoma, further mentioned as pancreatic cancer, is one of the most aggressive cancer with an overall 5-year survival of merely 4% in Western countries (http://seer.cancer.gov/csr/1975_2000/). It is described as a poorly differentiated tumour type that aggressively invades surrounding tissue and metastasises early to distant organs. Pancreatic cancer is usually undiagnosed until it has reached an advanced symptomatic stage, and consequently, only 15–20% of all patients are candidates for a surgical resection (Warren *et al*, 1983). Among those patients amenable to surgical resection, tumour characteristics including size and degree of differentiation are important independent prognostic factors (Sohn and Yeo, 2000).

A major characteristic of pancreatic cancers is an avascular morphology, which results in a poor blood and oxygen supply. Consequently, pancreatic tumours are generally hypoxic, which has been proven by intratumoral *p*O_2_ measurements (Koong *et al*, 2000). Hypoxia can contribute significantly to an aggressive behaviour of pancreatic cancers through the hypoxia-induced expression of proangiogenic factors, such as vascular endothelial growth factor (VEGF) and interleukin-8 (Shi *et al*, 1999; Buchler *et al*, 2003; Hotz *et al*, 2005). Tumour hypoxia also has been shown to increase tumour growth and the metastatic potential of pancreatic cancer cells (Niizeki *et al*, 2002; Buchler *et al*, 2004). Additionally, pancreatic cancers highly express the hypoxia-inducible transcription factor, hypoxia-inducible factor 1 (HIF-1) (Shibaji *et al*, 2003). HIF-1 is a heterodimeric protein that is tightly regulated through the oxygen-dependent degradation of its *α*-subunit. In the presence of oxygen, iron and 2-oxoglutarate HIF-1*α* protein is hydroxylated by a class of enzymes termed HIF prolyl hydroxylases. The hydroxylation leads to its rapid proteosomal degradation in a von Hippel Lindau protein-dependent manner (Wang and Semenza, 1993a; Salceda and Caro, 1997; Huang *et al*, 1998). Hypoxia can be mimicked by the heavy metal cobalt chloride (CoCl_2_), the iron-chelating agent, deferoxamine (DFX) or an inhibitor of the prolyl hydroxylases dimethyloxaloylglycine (DMOG), which all lead to the stabilisation of HIF-1*α* protein (Brahmachari and Joseph, 1973; Wang and Semenza, 1993b; Ivan *et al*, 2001). In tumours with a poor oxygen supply, HIF-1 target genes are induced, which help cell survival and encourage a more aggressive tumour phenotype by promoting growth, invasion and metastasis (Hockel *et al*, 1996; Vaupel *et al*, 2001; Yoon *et al*, 2001).

N-myc downstream-regulated gene 1 (NDRG1) is a recently described protein that is induced by cellular stress, in particular, it is significantly upregulated by hypoxia through HIF-1-dependent and -independent mechanisms (Park *et al*, 2000; Salnikow *et al*, 2000; Salnikow *et al*, 2002; Karaczyn *et al*, 2005). Other stress-inducing agents, such as homocysteine and tunicamycin (Kokame *et al*, 1996), nickel compounds (Zhou *et al*, 1998), synthetic retinoids (Piquemal *et al*, 1999) and compounds that lead to cellular differentiation (van Belzen *et al*, 1997) can also modulate its expression.

The precise biological function of NDRG1 is still not known, but various studies support its role as a potential tumour suppressor protein. Overexpression of NDRG1 *in vitro* results in morphological changes typical of cell differentiation and is inversely related to tumour growth and metastasis (Kurdistani *et al*, 1998; Guan *et al*, 2000; Bandyopadhyay *et al*, 2003; Bandyopadhyay *et al*, 2004a). Furthermore, its expression is increased by the tumour suppressor proteins p53 and PTEN (Kurdistani *et al*, 1998; Bandyopadhyay *et al*, 2004b) and clinical data have shown that its presence is statistically correlated with an increased survival of patients diagnosed with prostate cancer or liver metastasis from colorectal cancers (Bandyopadhyay *et al*, 2003; Shah *et al*, 2005).

NDRG1 mRNA is broadly expressed in many normal tissues, whereas its protein is generally restricted to cells of epithelial origin (Lachat *et al*, 2002). In some cancers, NDRG1 has been proposed to be a tumour marker because it is highly expressed in malignant compared to normal tissue of the same origin (Gomez-Casero *et al*, 2001; Nishie *et al*, 2001; Cangul *et al*, 2002b). However, the reliability of NDRG1 as a tumour marker is still undecided because its expression was initially shown to be repressed in colorectal cancer cells compared to the well-differentiated normal colon epithelial cells (van Belzen *et al*, 1997). Furthermore, NDRG1 is a highly stable protein and has been suggested as a prognostic marker for hypoxic regions within a tumour mass (Park *et al*, 2000; Cangul *et al*, 2002b; Lachat *et al*, 2002).

Thus far, NDRG1 expression has yet to be studied in pancreatic cancer. We hypothesised that NDRG1 may be a novel indicator of malignant cells in the pancreas as hypoxia is a general feature of these tumours. The data presented in this study demonstrate that regardless of the hypoxic environment, there is a selective expression of NDRG1 in the pancreatic tumour cells. Therefore, we suggest that the determining factor of NDRG1 expression in pancreatic cancer is not hypoxia, but rather the differentiation status of the tumour.

## MATERIALS AND METHODS

### Immunohistochemistry

Human pancreatic samples were obtained at our institution from consented patients by standard surgical oncology procedures. Tissue samples from patients diagnosed with pancreatic cancer (*n*=27) and pancreatic intraepithelial neoplasia (PanIN) (*n*=6) were compared to samples taken from tumour-free resection margins (*n*=11) and normal tissues obtained by organ donation or from patients with nonpancreatic diseases (*n*=7). Formalin-fixed samples were paraffin embedded and cut into 5 *μ*m thin sections for analysis. Deparaffinised sections were microwave heated for 20 min in 0.01 M Na-citrate, pH 6.4, for antigen retrieval. Endogenous peroxidases were blocked by 0.3% H_2_O_2_ in methanol for 30 min. Sections were incubated with either affinity-purified polyclonal rabbit anti-NDRG1 antibody (1:100, Zymed® Laboratories Inc., 52-3557, San Francisco, CA, USA), a polyclonal affinity-purified antibody raised in sheep against the full-length protein (1:3000, Kinasource AB-160 Limited, Scotland, UK) or with a monoclonal antibody against HIF-1*α* (clone H1alpha67-sup; Novus Biologicals Inc., Colorado, USA) overnight at 4°C. Specificity of the NDRG1 antibody from Zymed® was confirmed by blocking with the peptide immunogen. Sections were further developed with components of the Vectastain® Kit (Vector Laboratories Inc., Burlingame, CA, USA) according to the manufacturer's instructions. Immunoreactivity was developed using 3,3′-diaminobenzidine as the peroxidase substrate and nuclei were counterstained with haematoxylin. Negative controls were performed by substituting the first antibody with rabbit IgG (Dako, Schweiz AG, Baar, CH). For histological analysis, serial sections were stained with haematoxylin and eosin and documented using a Leica DMRB microscope with IM50 Leica imaging software. The samples were evaluated by a pathologist (RW) and classified according to Klöppel's grading (Klöppel *et al*, 2000). In brief, G1 (*n*=3) was stated in samples bearing well-differentiated duct-like glands, intensive mucin production, <5 mitoses per 10 high-power fields (HPF), little nuclear polymorphism or polar arrangement. G2 (*n*=15) was stated in samples bearing moderately differentiated duct-like and tubular glands, irregular mucin production, 6–10 mitosis per 10 HPF and moderate nuclear polymorphism. G3 (*n*=9) was stated in samples bearing poorly differentiated glands, mucoepidermoid and pleomorphic structures, abortive mucin production, >10 mitosis per 10 HPF and marked nuclear polymorphism and increased nuclear size. The intensity of immunohistochemical staining was scored 0 for no staining or from 1 to 3, if cells were positively stained in more than 10% of the tumour. A score of 1 corresponded to weak and partial cell staining, 2 represented weak and complete staining and 3 for strong and complete staining. The mean intensity of 5 HPF per section was calculated.

### Cell lines and culture conditions

Human pancreatic cancer cells Capan-1 (moderately differentiated) and Panc-1 (poorly differentiated) were purchased from ATCC (LCG Promochem, Molsheim, France) and cultured in Dulbecco's modified Eagle's medium supplemented with 20% heat-inactivated fetal bovine serum for Capan-1 and 10% for Panc-1, 100 U/ml penicillin and 100 *μ*g/ml streptomycin (all from Life Technology, Paisley, Scotland) and incubated at 37°C in a humidified atmosphere with 5% CO_2_. Hypoxic culture conditions were performed in a microaerophilic system (Ruskinn, Biotrace International, Bridgend, UK) at 2% O_2_, 5% CO_2_ and 93% N_2_ for 2, 4, 12, 24 and 48 h. To test additional activators of HIF-1*α*, cells were exposed to 125 *μ*M DMOG (Alexis Biochemicals, Lousen), 100 *μ*M CoCl_2_ (Sigma, Bucks, CH) or 100 *μ*M DFX (Sigma, Bucks, CH).

### RNA extraction and real-time PCR

Total RNA was extracted with TRIzol® (Life Technologies, Paisley, Scotland) according to the manufacturer's instructions. One microgram of total RNA was DNase treated (Promega, Madison, WI, USA) and reverse transcribed into cDNA with a commercial kit (Qiagen, Hilden, Germany). FAM™ dye-labelled TaqMan® MGB probes and polymerase chain reaction (PCR) primers were purchased for human NDRG1 and VEGF at Applied Biosystems (Warrington, UK). As internal positive control, 18S was used with a VIC® dye-labelled TaqMan® MGB probe. Real-time PCR was performed using ABI PRISM™ 7000 Sequence Detector System (Applied Biosystems). The amplification conditions were as follows: 40 cycles at 95°C for 15 s (denaturation step) and 60°C for 1 min (combined annealing–extension step). Each experiment was carried out in triplicate. Mean cycle threshold (*C*_t_) values were calculated for 18S and the reporter gene. *C*_t_ values for NDRG1 and VEGF were normalised against the internal ribosomal RNA (18S) control probe to calculate Δ*C*_t_ values. ΔΔ*C*_t_ values were calculated by subtracting the Δ*C*_t_ values of cells under normoxia from the Δ*C*_t_ value of cells under hypoxia. Fold increase was calculated using the formula 2^−(ΔΔC^_t_). Each experiment was repeated three times. After three repetitions mean and s.d. were calculated.

### Protein extraction and Western blotting

For NDRG1 analysis, total cell lysates were prepared using 10 mM Tris (pH 8.0), 1 mM EDTA (pH 8.0), 150 mM NaCl and 0.5% NP-40 with the inhibitors 1 mM NaF, 10 mM Na_3_VO_4_, 1 mM PMSF and 1 × protease inhibitor cocktail (P-8340, Sigma). For HIF-1*α*, nuclear-enriched protein extracts were prepared as described previously (Jewell *et al*, 2001). Total protein concentrations were determined by Bio-Rad Protein Assay (Bio-Rad, Reinach, CH) and equal amounts (20 *μ*g) were separated by sodium dodecyl sulphate–polyacrylamide gel electrophoresis (10%). Proteins were transferred to nitrocellulose membrane using a semidry transfer system (Bio-Rad), blocked with 5% nonfat dry milk in 50 mM Tris (pH 7.5), 150 mM NaCl, 0.1% Tween-20 and incubated with the primary antibody (either sheep anti-NDRG1 (0.1 *μ*g/ml, Kinasource AB-160, Scotland) or mouse anti-HIF-1*α* (1:500, courtesy of Professor Max Gassmann, Zürich Switzerland)), rabbit anti-*β*-actin (1:500, Sigma A5060) or rabbit anti-Sp1 (Santa Cruz, SC-59, California, USA) overnight at 4°C. Membranes were incubated with either anti-sheep (1:3000, Dako), anti-mouse (1:10 000, Pierce) or anti-rabbit (1:10 000, Dako) HRP-labelled secondary antibodies for 1 h at room temperature and developed with an enhanced chemiluminescent substrate (LiteAblot, Euroclone SpA, Lugano, Switzerland).

### Statistics

Immunohistochemical data were analysed with the Mann–Whitney *U*-test. For the comparison of the real-time PCR data, Δ*C*_t_ values were analysed by one-way analysis of variance (ANOVA) followed by Dunnett's multiple comparisons test. The statistical analysis software, SPSS (version 11.5), was used and *P*<0.05 was considered significant.

## RESULTS

### NDRG1 protein is expressed in peritumoural, hyperplastic pancreatic areas

**Figure 1:**
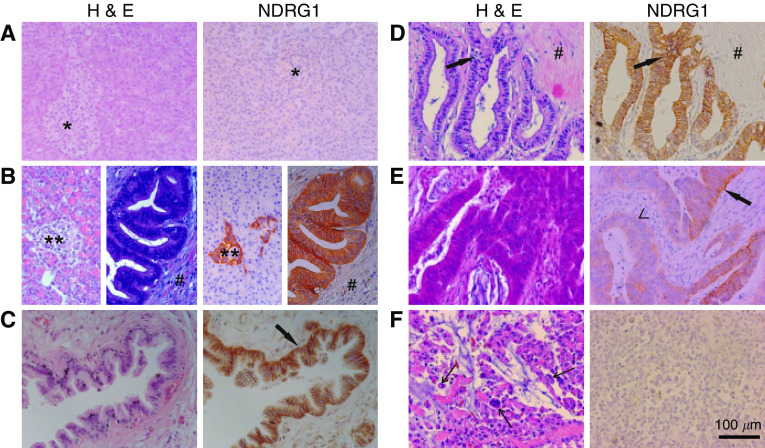
Photomicrographs of haematoxylin and eosion staining and NDRG1 immunohistochemistry of pancreatic tissue sections (original magnification × 200). (**A**) Normal pancreatic tissue from organ donors, with the pancreatic islets (*) surrounded by acini. (**B**) Peritumoral tissue with hyperplastic islets (**), hyperplastic ducts and fibrotic peritumoral tissue (#). (**C**) PanIN of the main duct ( → ). (**D**–**F**) Ductal adenocarcinomas of the pancreas, (**D**) well-differentiated tumour ( → ), embedded in the tumour stroma (#). (**E**) Moderately differentiated tumour with strongly staining malignant cells ( → ), beside malignant cells showing a faint staining (<). (**F**) Undifferentiated tumour characterised by polynuclear cells ←.

NDRG1 protein expression was not found in unmodified pancreatic tissue obtained from organ donors; specifically, there was no staining in the glandular epithelial cells of the acini or in the pancreatic islet cells (Figure 1A). However, pancreatic tissue obtained from tumour-free resection margins displayed strong staining for NDRG1 specifically in hyperplastic islets and hyperplastics ducts (Figure 1B). The peritumoral tissue is nonquiescent and considered reactive because it is under the influence of stimulating factors secreted by the tumour. The observed staining in these areas demonstrates that NDRG1 is expressed in nontumoral reactive pancreatic tissue.

### NDRG1 protein as a marker of pancreatic cancer differentiation

**Figure 2:**
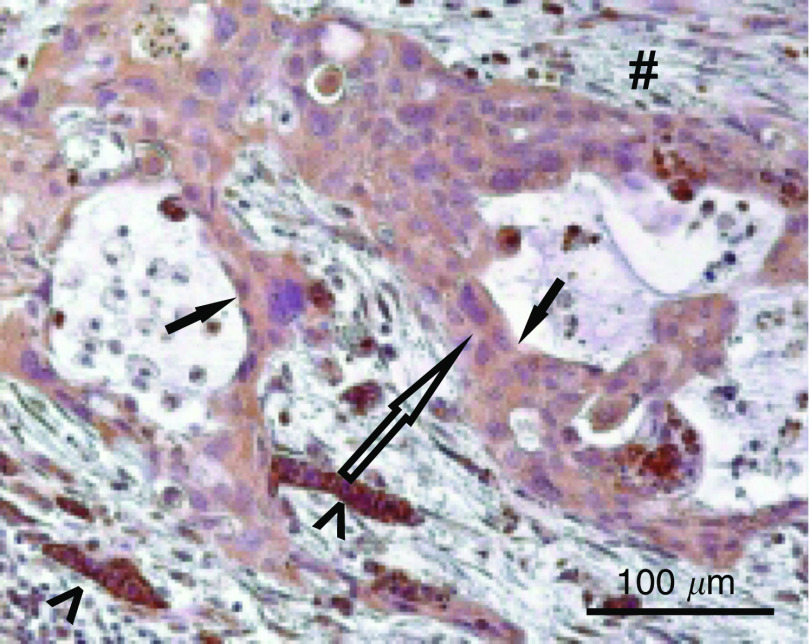
Photomicrograph of NDRG1 immunohistochemistry (original magnification × 200) of a moderately differentiated ductal adenocarcinoma of the pancreas → , surrounded by fibrotic tissue (#), with embedded regenerating tubular complexes (<). 
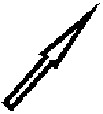
 The possible pathomechanism of dedifferentiation.

Samples from patients diagnosed with pancreatic cancer were graded G1–3 (Klöppel *et al*, 2000) or as a PanIN. In all the samples, NDRG1 staining was restricted to the tumour epithelial cells and no staining was visible in fibrocytes of the desmoplastic reaction. In PanIN (Figure 1C) and in well-differentiated tumour regions (G1, *n*=3) (Figure 1D), there was a distinct staining of the epithelial cells. Moderately differentiated tumour regions (G2, *n*=15) showed a less prominent expression with patchy and irregular patterns on the membranes (Figure 1E). The median intensity score of G2 graded tumours was 1.8 with an interquartile range of 1.3–2.0. In comparison, there was nominal staining for NDRG1 in tumours that lost histopathological signs of differentiation (G3, *n*=9) (Figure 1F). The median intensity score of G3 graded tumours was 0.8 with an interquartile range of 0.6–1.4. This was significantly lower than in G2 graded tumours (*P*=0.014). Furthermore, on resection margins it was frequently noted that, nonmalignant, highly differentiated tubular complexes in reactive pancreatic areas showed a stronger staining than the adjacent tumour cells (Figure 2).

**Figure 3:**
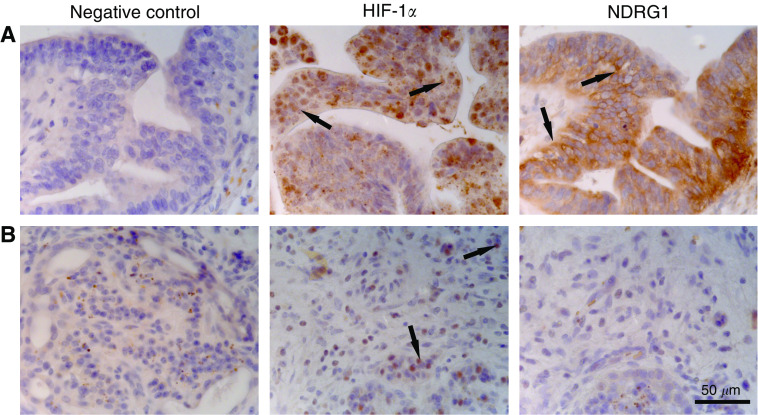
Photomicrographs of (**A**) moderately differentiated and (**B**) poorly differentiated ductal adenocarcinomas of the pancreas stained for HIF-1*α* and NDRG1 (original magnification × 400). ( → ) Indicating positive reactivity in tumour cells.

Cellular hypoxia is a characteristic feature of pancreatic tumours and is generally known to act as potent inducer of NDRG1 expression. Using HIF-1*α* expression as a marker for tumour hypoxia, we demonstrate that in both differentiated and undifferentiated pancreatic tumours HIF-1*α* protein is stabilised. As expected, immunoreactivity for HIF-1*α* was observed in the nuclei of tumour cells (Figure 3). In serial sections, NDRG1 was colocalised only in well-differentiated tumour regions.

### Hypoxia-induced upregulation of NDRG1 is influenced by pancreatic cancer cell differentiation

**Figure 4:**
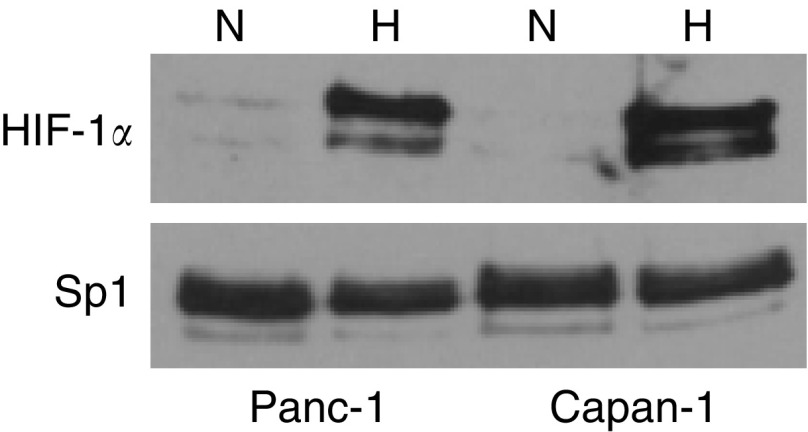
HIF-1*α* protein is stabilised in Capan-1 and Panc-1 cells under hypoxia. Nuclear enriched protein extracts from cells cultured either under normoxia (N) or hypoxia (H: 2% O_2_) for 24 h. Proteins were analysed by Western blot using a monoclonal antibody against HIF-1*α* (120 kDa) or a rabbit polyclonal antibody against Sp1 (87 kDa) to control for equal nuclear protein loading. Signals were developed with enhanced chemiluminescence.

**Figure 5:**
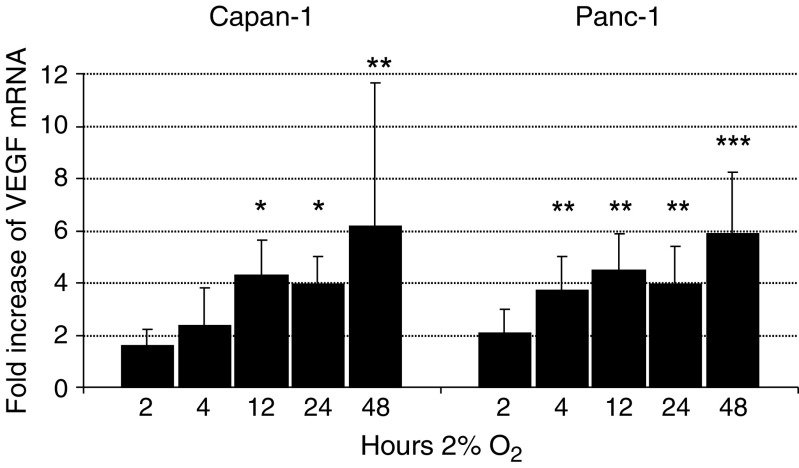
VEGF mRNA is equally upregulated in both Capan-1 and Panc-1 cells under hypoxia. Cells were grown under hypoxic conditions (2% O_2_) for 2, 4, 12, 24 and 48 h. The bars represent the mean fold increase plus s.d. of hypoxic cells compared to normoxic controls. Results are from three independent experiments. Statistics were calculated by ANOVA followed by Dunnett's multiple comparisons test, ^*^P<0.05, ^**^P<0.01, ^***^P<0.001.

To test whether hypoxia-induced expression of NDRG1 is dependent on differentiation, two pancreatic cancer cells lines, the moderately differentiated Capan-1 and the poorly differentiated Panc-1, were cultured either at 21% O_2_ (normoxia, N) or at 2% O_2_ (hypoxia, H). Exposure to 2% O_2_ is sufficient to stabilise the *α*-subunit of HIF-1 protein (Figure 4). HIF-1*α* is stabilised and strongly detected by Western blot in nuclear protein extracts from cells cultured at 2% O_2_ compared to normoxic controls in both tumour cell lines. Equal amounts of protein were controlled for with Sp1. VEGF mRNA expression was examined by real-time PCR as a control for the induction of an established HIF-1-dependent gene. Both cell lines show a significant 4–6-fold increase of VEGF mRNA after 12 h that was maintained up to 48 h of exposure to hypoxia (Figure 5).

**Figure 6:**
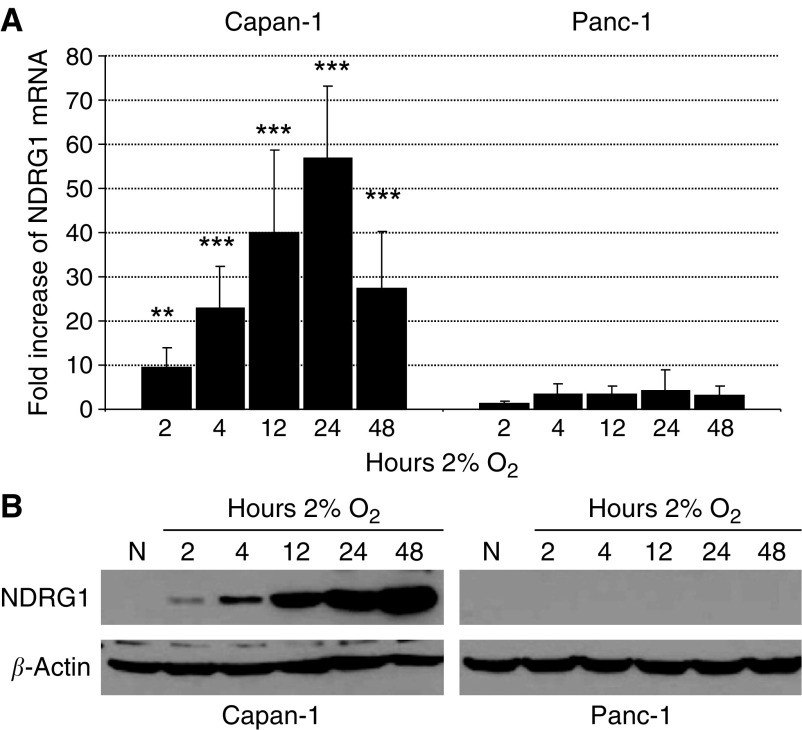
Comparison of NDRG1 mRNA (**A**) and protein (**B**) expression in Capan-1 and Panc-1 cell lines. (**A**) Refer to Figure 5 for explanation. (**B**) Cells were exposed to normoxia (N) or hypoxia (2% O_2_) for 2, 4, 12, 24 or 48 h. Total protein lysates were analysed by Western blot with a sheep anti-NDRG1 antibody (43 kDa). Signals were developed with enhanced chemiluminescence. *β*-actin (42 kDa) is shown as a control for equal protein loading.

Capan-1 and Panc-1 cells constitutively express similar levels of NDRG1 mRNA under normoxic conditions (data not shown). Under hypoxia, however, NDRG1 mRNA was upregulated 10-fold after 2 h and steadily increased reaching a 60-fold induction after 24 h in the moderately differentiated Capan-1 cells. In contrast, there was no significant increase of NDRG1 mRNA at any time point tested in the poorly differentiated Panc-1 cells (Figure 6A). By Western blot, there was no expression of NDRG1 protein in both cell lines cultures under normoxic conditions. Yet, consistent with the mRNA analysis, NDRG1 protein was detected after 2 h of hypoxia and its level steadily increased reaching a maximum expression after 48 h in Capan-1 cells. There was no NDRG1 protein expression at all time points tested in Panc-1 cells. Equal amounts of protein were controlled for with *β*-actin (Figure 6B).

**Figure 7:**
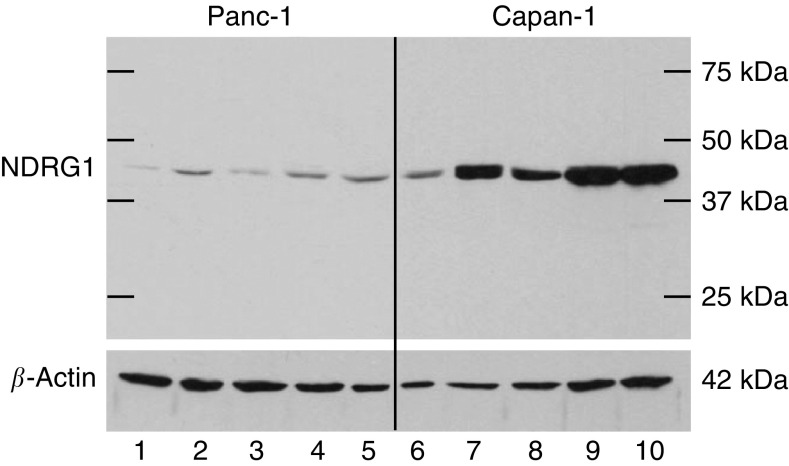
Comparison of NDRG1 expression in Panc-1 and Capan-1 cell lines exposed to HIF-1*α* activators. Panc-1 and Capan-1 cell lines were exposed to several HIF-1*α* activators for 24 h. Total protein lysates were analysed by Western blot with a sheep anti-NDRG1 antibody (43 kDa). *β*-Actin (42 kDa) is shown as a control for equal protein loading. Lanes 1 and 6: normoxia; lanes 2 and 7: 125 *μ*M DMOG; lane 3 and 8: 100 *μ*M cobalt chloride; lanes 4 and 9: 100 *μ*M DFX; and lanes 5 and 10: hypoxia with 2% O_2_.

To confirm that NDRG1 expression is dependent on the cellular differentiation status of cells, other activators of HIF-1*α* were tested. There was a strong induction of NDRG1 protein in Capan-1 cells incubated with DMOG, CoCl_2_, DFX and under hypoxia in comparison to Panc-1 cells, at equal loading and exposure time (Figure 7). Analysis of NDRG1 mRNA confirmed these results (data not shown). Of note, unlike in the previous Western blots shown, we found faint signals in the Panc-1 cells, which are a result of increased antibody sensitivity.

Taken together, this series of experiments suggest a selective loss of NDRG1 expression despite HIF-1*α* stabilisation in the undifferentiated pancreatic cell line, Panc-1.

## DISCUSSION

NDRG1 is strongly induced by hypoxia, one of the key characteristics of pancreatic cancer, thus our first goal was to establish NDRG1 as a tumour marker for this aggressive malignancy. Initially, our data in the pancreas were consistent with previous reports on other cancers (Gomez-Casero *et al*, 2001; Nishie *et al*, 2001; Cangul *et al*, 2002a), showing that NDRG1 protein is highly expressed in tumours compared to normal tissue of the same origin. However, pancreatic cancers induce a strong peritumoral desmoplastic reaction characterised by fibrosis and hypoxia. Within this region, where pancreatic regeneration is an active process, we observed a strong staining of newly formed, highly differentiated tubular complexes as well as in hyperplastic ducts and islets. The high NDRG1 expression levels found in these reactive areas supports studies that have demonstrated its modulation during differentiation and by multiple stress-inducing agents.

On closer examination, we observed that PanIN and areas consisting of well-differentiated cells were strongly stained within pancreatic tumours. Conversely there was very little to no expression of NDRG1 protein in poorly differentiated cells, although we showed HIF-1*α*-positive nuclei in these regions. This observation was in agreement with studies of colorectal and prostatic cancer, in which NDRG1 was reduced in poorly differentiated adenocarcinoma compared to well-differentiated cells (van Belzen *et al*, 1997; Guan *et al*, 2000; Caruso *et al*, 2004). The difference being that, quiescent apical colonic epithelia express NDRG1, whereas normal pancreatic cell types do not.

From a clinical point of view, the observed presence of NDRG1 in nontumoral tissue taken together with its negligible expression in undifferentiated tumours argues against its value as a reliable tumour marker. It may, however, be a potential marker to predict the differentiation status of pancreatic cancer cells. Within a tumour lesion, loss of expression would serve as a poor prognostic indicator and a tool to plan the adjuvant therapy. In a retrospective analysis, high levels of NDRG1 was associated with indolent tumour growth and with improved survival in patients diagnosed with colorectal cancer (Shah *et al*, 2005).

As pancreatic cancer is characterised as a hypoxic tumour, we next questioned why there was negligible NDRG1 expression in the undifferentiated tumour cells. Hypoxia can influence cellular phenotypes by altering the expression of specific genes and is generally thought to give pancreatic cancers cells an advantage by promoting factors beneficial for tumour growth and survival (Duffy *et al*, 2003). This idea is maintained by our *in vitro* experiments in which HIF-1*α* protein and VEGF mRNA were upregulated by hypoxia in both cell lines tested. Whereas Panc-1, the more aggressive undifferentiated cell line, showed a modest increase of NDRG1 mRNA or protein under different HIF-1*α* stabilizer, compared to the prominent response of the moderately differentiated tumour cell line, Capan-1. This suggests that the expression of NDRG1 may be dependent on cell differentiation. If indeed NDRG1 is a potential tumour suppressor protein, it would be a survival advantage for an aggressive cancer to shut down the ability to upregulate a growth inhibitory protein. The loss of ability to express NDRG1 would assist cells to dedifferentiate to a more aggressive phenotype with more metastatic potential. Recently, it has been suggested that hypoxia can induce dedifferentiation and genetic instability of tumour cells, which may account for the heterogeneity and aggressiveness of solid tumours (Reynolds *et al*, 1996; Jogi *et al*, 2002; Helczynska *et al*, 2003; Unruh *et al*, 2003).

NDRG1 is a very stable protein and has been proposed as an indicator of tumour hypoxia (Cangul *et al*, 2002a). From our studies, along with the published work of others, we would predict that its use as a marker of hypoxia would also be unreliable. The first point, as we suggest here, its expression is dependent on the differentiation status of cells within a hypoxic environment. Furthermore, other potential markers such as HIF-1*α* and its target genes Glut-1 and CAIX were proven unreliable because of the influence of other regulating factors (Mayer *et al*, 2004, 2005a, 2005b). In addition to oxygen deprivation, hypoxia-regulated genes can be influenced by nutrient deficiencies, oncogenic mutations and oxidative stress. NDRG1 expression is also modulated by ascorbate levels within a tumour microenvironment, directly demonstrating an alternative mechanism of its regulation (Karaczyn *et al*, 2005).

In this study, our data suggest that it is the differentiated cells that can express NDRG1 in a hypoxia-stressed environment. Hypoxic areas within solid tumours are diverse and nonuniform through the new formation and collapse of subfunctional blood vessels. This may account for the observed patchy pattern of NDRG1 expression we observed in moderately differentiated tumours. The mechanism by which this response is lost in undifferentiated cells warrants further investigations.

Pancreatic cancer is characterised by its predisposition to aggressively invade surrounding tissues, to metastasise early and extensively and to resist conventional chemoradiation treatment strategies. Although the precise function of NDRG1 is unknown, there is solid evidence that suggests it may suppress the invasive ability and spontaneous metastasis of cancer cells by inducing differentiation and reversing a metastatic phenotype. Further studies are aimed to clarify the function of this novel protein, and its role in the progression and metastasis of various cancers. Clinically important, the data provided suggest that NDRG1 may offer a powerful diagnostic tool for the grading of pancreatic cancers, whereas better characterising of the protein may help to decide on how aggressive the adjuvant therapy should be planned.
